# Amygdala–hypothalamus–brainstem circuits underlying cardiovascular responses associated with the limits of high-intensity endurance exercise

**DOI:** 10.3389/fphys.2025.1714093

**Published:** 2026-01-12

**Authors:** Ko Yamanaka, Jimmy Kim, Kei Tsukioka, Shinichiro Ezure, Hiroyasu Ichihara, Linh Thuy Pham, Hidefumi Waki

**Affiliations:** 1 Graduate School of Health and Sports Science, Juntendo University, Chiba, Japan; 2 Institute of Health and Sports Science and Medicine, Juntendo University, Chiba, Japan

**Keywords:** amygdala, blood pressure, high-intensity endurance exercise, hypothalamus, medulla, sympathetic nervous system

## Abstract

In athletic competitions, athletes continually challenge the limits of human performance. Exercise limitation refers to a state in which fatigue accumulates during prolonged activity, preventing the maintenance of the required power output despite maximal voluntary effort. High-intensity endurance exercise compromises muscle performance due to the accumulation of metabolic by-products in the peripheral tissues. Sympathetic nerve activation during exercise increases blood flow to the working muscles and aids in fatigue-inducing substance removal. However, excessive sympathetic activity may lead to peripheral muscular vasoconstriction, limiting exercise capacity. The present review explored the roles of the central autonomic regions, including the central nucleus of the amygdala (CeA), the paraventricular nucleus of the hypothalamus (PVN), and nucleus tractus solitarii (NTS) of the medulla in endurance limitation. The CeA is selectively activated during high-intensity exercises and contributes to the sympathetic drive. CeA lesions prolong exercise duration and delay blood pressure surges before exhaustion, suggesting that the CeA may act as a central “brake” on performance. Moreover, the co-activation pattern of the CeA–PVN–NTS circuits appears to shift dynamically depending on the exercise intensity. Understanding this emotion–autonomic circuits may provide new insights into exercise limitation and suggest novel strategies for enhancing endurance performance.

## 1 Introduction

In competitive sports, athletes continuously push the boundaries of human performance, making the understanding of the physiological and psychological limits, referred to as exercise limitation (Jones and Killian, 2000), one of the central issues in sports science. Especially in high-intensity and endurance exercises, such as marathon running and cycling, various factors, including muscle fatigue, oxygen supply capacity, energy metabolism, and neuronal control function, are related, leading to a state of “exhaustion” in which exercise stops.

The mechanisms that define exercise limitations have been broadly classified into central and peripheral fatigue. Central fatigue refers to a fatigue originating in the central nervous system, particularly in the cerebral cortex and subcortical structures, and is caused by reduced output of motor commands necessary for movement execution. Contrarily, peripheral fatigue refers to a condition in which the output is physically limited due to the functional decline in the muscles, neuromuscular junctions, and other motor units themselves (Gandevia, 2001).

Traditionally, maximal oxygen uptake (VO_2_max) has been emphasized in endurance performance evaluation. However, considerable performance differences are observed even among athletes with comparable VO_2_max (Coyle et al., 1988), suggesting that oxygen supply capacity alone may not be sufficient to explain exercise limitations. Moreover, extreme hypoglycemia during endurance exercise leads to a rapid decline in performance (hunger knock), but, conversely, there are reports that maintaining high blood glucose levels during exercise does not necessarily improve performance (Hargreaves et al., 1987). Furthermore, the individuals who have reached exhaustion are still capable of producing high muscle output, suggesting that “exercise limitation” may not be determined by the mechanical limits of the muscle itself, but rather by the perception of effort (Marcora and Staiano, 2010).

In the present review, we focused on “central fatigue,” particularly on the subcortical neural mechanisms involved in autonomic regulation, to discuss the neuronal basis of exercise limitation. Central fatigue has often been attributed to the activity of the primary motor cortex and spinal motor neurons; however, this review focused on the subcortical networks involved in cardiovascular regulation and emotional control, specifically the amygdala–hypothalamus–brainstem medulla oblongata. These structures regulate the cardiovascular system, including blood pressure, heart rate, and blood flow, via the sympathetic premotor and parasympathetic neurons, which not only contribute to homeostatic control at rest but also may influence allostatic control during exercise. Here, we outline the neural basis of central fatigue during high-intensity endurance exercise, such as marathon running, drawing primarily on recent findings from animal models, including those from our own laboratory, to explore the neuronal mechanisms underlying exercise limitation.

## 2 Medulla oblongata, amygdala, and hypothalamus as central cardiovascular regulatory centers

During exercise, the activation of the sympathetic nervous system (Miki et al., 2002) elicits cardiovascular responses, including increased myocardial contractility and peripheral vasoconstriction, thereby elevating the blood pressure, heart rate, and muscle blood flow. Enhanced sympathetic activity leads to blood vessel constriction throughout the body, including the skeletal muscle, thereby increasing peripheral vascular resistance. However, contraction-induced release of metabolic by-products, including nitric oxide (NO), prostaglandins, adenosine, lactate, and potassium ions, induces local vasodilation. As a result, the total peripheral resistance decreases and blood flow to the active muscles increases (Rowell, 1974; Clifford and Hellsten, 2004). During high-intensity endurance exercises approaching the limit of tolerance, excessive sympathetic activation may suppress these vasodilatory responses, potentially compromising oxygen delivery to the active muscles. The integration of such cardiovascular control during exercise is mediated primarily by brainstem’s autonomic centers, particularly the medulla oblongata, and elucidating the function of these neural circuits is essential for understanding the mechanisms underlying exercise limitation.

The nucleus tractus solitarii (NTS), located in the dorsal medulla, receives afferent inputs from arterial baroreceptors, chemoreceptors, and muscle metaboreceptors, functioning as a central hub for cardiovascular reflexes, including the baroreceptor and exercise pressor reflexes (Michelini, 2007; Kaufman, 2012). NTS regulation based on these ascending inputs is regarded as feedback control, adjusting autonomic output in response to the changes in blood pressure, metabolic by-products, and other physiological variables. Contrarily, the NTS also receives descending central command signals from the higher brain centers, enabling feedforward control that initiates predictive adjustments in the autonomic activity prior to exercise onset (Michelini and Stern, 2009; Michelini et al., 2015). Such feedforward inputs allow predictive autonomic regulation in response to the intention and planning of arbitrary motor execution (Ishii et al., 2016).

These ascending and descending signals integrated in the NTS are transmitted to the rostral ventrolateral medulla (RVLM) via the inhibitory neurons of the caudal ventrolateral medulla (CVLM) located in the ventral medulla. The RVLM contains sympathetic premotor neurons that project directly to the sympathetic preganglionic neurons in the spinal cord, thereby regulating cardiac output and peripheral vascular resistance. Thus, the NTS is considered to play a central role in modulating cardiovascular responses during exercise by integrating both feedback and feedforward control mechanisms (Waki, 2012).

Descending inputs to the NTS originate from higher brain regions such as the hypothalamus and limbic system. The paraventricular nucleus of the hypothalamus (PVN) contains sympathetic premotor neurons that directly regulate the sympathetic preganglionic neurons in the spinal cord (Dampney, 1994; Guyenet, 2006) and indirectly control cardiovascular function via projections to the midbrain and medullary nuclei (Koba et al., 2018; Koba et al., 2022). During exercise, in addition to its neuroendocrine output (e.g., vasopressin and oxytocin), the PVN sends projections to the medullary regions involved in sympathetic regulation, such as the NTS and RVLM, thereby modulating the gain of cardiovascular reflexes (Michelini and Stern, 2009).

The amygdala, which is a key component of the limbic system, is well known as an emotional regulation center; however, it is also involved in autonomic control. The amygdala comprised several subnuclei (Swanson and Petrovich, 1998; Sah et al., 2003), but the central nucleus of the amygdala (CeA) is the main output nucleus and has descending projections to the PVN, NTS and RVLM (van der Kooy et al., 1984; Saha, 2005; Zhang et al., 2021; Shao et al., 2024). Moreover, the stimulation and manipulation of CeA elicit cardiovascular responses including increased blood pressure and heart rate (Yamanaka et al., 2018; Sheng et al., 2022).

Thus, the NTS in the medulla oblongata, which is the brainstem’s cardiovascular regulatory system, may receive higher-level inputs, including PVN and CeA, and regulate cardiovascular responses during exercise (Potts, 2006; Michelini and Stern, 2009).

## 3 Amygdala responses during high-intensity endurance exercise

The effects of exercise on emotion are dose-dependent; low-intensity exercise induces positive emotional responses (Salmon, 2001), whereas high-intensity endurance exercise approaching exhaustion often triggers negative emotional responses (Ekkekakis and Petruzzello, 1999). Among the brain regions involved in stress and emotional processing, the amygdala is a key center (LeDoux, 2007; Roozendaal et al., 2009; Zhang et al., 2021; Yamanaka and Waki, 2022).

**FIGURE 1:**
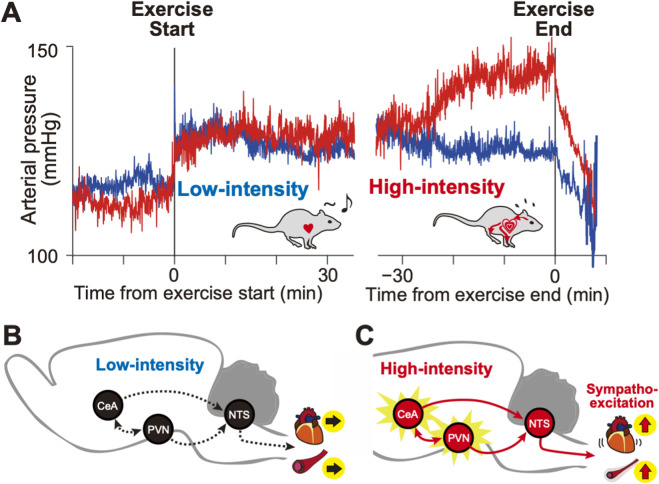
Differential cardiovascular responses to low- and high-intensity exercise in rats. **(A)** Low-intensity exercise was associated with relatively stable arterial pressure (blue), whereas high-intensity exercise induced a progressive elevation of arterial pressure (red) toward the exercise termination. Arterial pressure data are adapted from Kim et al. (2020). **(B,C)** Schematic representation of the CeA–PVN–NTS pathway during low-intensity **(B)** and high-intensity exercise **(C)**. During high-intensity exercise, enhanced activation of this pathway elicits a pressor response via sympathetic excitation.

Using a treadmill running model in rats, Kim et al. (2020) examined the impact of high-intensity endurance exercise on the activity of the amygdala by quantifying c-Fos expression as a neuronal activity marker (Kim et al., 2020). Male Wistar rats were assigned to the following three groups: 1) sedentary (placed on the treadmill without running), 2) low-intensity exercise (20 m/min for 45 min), and 3) high-intensity exercise (34 m/min for 90 min or until exhaustion). All animals were implanted with telemetry transmitters in the abdominal aorta to record their blood pressure, heart rate, and body temperature during exercise. The cardiovascular parameters showed distinct patterns. The heart rate increased proportionally to the running speed, whereas the blood pressure and body temperature increased with the onset of exercise and then remained at constant levels. In the high-intensity exercise group, however, both blood pressure and body temperature exhibited a sharp increase at approximately 20–30 min before exercise offset, including at the point of exhaustion (Figure 1A). Immunostaining revealed considerably increased c-Fos expression in the basolateral amygdala (BLA) and CeA of the amygdala in the high-intensity exercise group. These expressions increased in an exercise intensity-dependent manner and were almost absent in the low-intensity exercise and sedentary groups. This data suggest that CeA is selectively activated during high-intensity endurance exercise, which is presumably accompanied by negative emotional states.

To directly investigate CeA’s involvement in cardiovascular regulation, electrical or chemical stimulation (disinhibition) with the gamma-aminobutyric acid receptor antagonist bicuculline of the CeA in urethane-anesthetized rats induced considerable increases in blood pressure and heart rate when applied to medial subdivision of CeA, which contains a high density of projection neurons (Yamanaka et al., 2018). These results support the notion that the CeA serves as a neural substrate capable of modulating the cardiovascular system through enhanced sympathetic outflow.

Taken together, these findings suggest that the amygdala, particularly the CeA, is activated under extreme stress during high-intensity endurance exercise, such as when approaching the exercise limit, and may regulate cardiac output and blood pressure via the sympathetic nervous system. Although this function of the CeA may help sustain performance to some extent, excessive sympathetic activation can induce skeletal muscle vasoconstriction, thereby restricting blood flow and preventing exercise continuation. In other words, the CeA may act within the central nervous system as a “brake” on exercise performance or a trigger for exercise termination.

## 4 Changes in endurance performance associated with amygdala lesion

To test the possibility that CeA may function as a central “brake” that defines exercise limits during high-intensity endurance exercise, Tsukioka et al. (2022) evaluated the treadmill running performance and cardiovascular responses during high-intensity exercise in rats in which the bilateral CeA was electrically lesioned (Tsukioka et al., 2022). After progressively increasing the treadmill running speed (incremental exercise test), the maximum exercise duration was considerably longer in the CeA-lesioned rats than in the sham-operated rats. Additionally, as a cardiovascular response, the blood pressure increased at the start of exercise, and a rapid rise in blood pressure appeared just before exhaustion as the exercise continued; this phenomenon is similar to that observed in a previous study (Kim et al., 2020). However, in CeA-lesioned rats, the timing of the onset of rapid blood pressure increase was considerably delayed, suggesting that CeA function is involved in the cardiovascular response during exercise and influences the timing of reaching exhaustion.

To determine whether the CeA neurons activated by high-intensity endurance exercise project to the medullary NTS, rats were pre-injected with a retrograde tracer (cholera toxin subunit B) into the NTS and then subjected to incremental exercise test. When the brain tissue was removed and analyzed after exercise, c-Fos expression in the CeA neurons projecting to the NTS (CeA→NTS neuron) and hypothalamic PVN neurons projecting to the NTS (PVN→NTS neuron) was markedly increased after the high-intensity exercise. These results indicate that the pathways from both CeA and hypothalamic PVN to the medullary NTS are activated during a high-intensity exercise.

## 5 Co-activation of the CeA and hypothalamic PVN and cardiovascular responses

To assess the functional impact of the CeA and PVN on cardiovascular regulation, electrical stimulation was applied to these regions individually or simultaneously in urethane-anesthetized rats, while the arterial blood pressure, muscle blood flow, and muscle vascular resistance (calculated as blood pressure divided by the muscle blood flow) were measured (Tsukioka et al., 2022). The stimulation of either the CeA or PVN alone considerably increased both blood pressure and muscle blood flow, without markedly altering the muscle vascular resistance. When comparing the results of CeA and PVN stimulations alone, PVN stimulation was found to produce a greater increase in muscle blood flow than the CeA stimulation. Importantly, simultaneous CeA and PVN stimulations elicited not only a considerable greater pressor response but also a robust and marked increase in muscle vascular resistance, an effect that was not observed in either site alone. Although muscle blood flow also increased under simultaneous stimulation, the magnitude of this increase did not differ significantly from that of individual stimulation.

Taken together, these findings support the idea that the CeA and PVN function as feedforward control centers for cardiovascular regulation during exercise. The simultaneous excitation of the CeA and PVN appears to restrict blood flow to the skeletal muscle by increasing vascular resistance. Given that the CeA shows minimal activation during low-intensity exercises and strong activation during high-intensity exercises (Kim et al., 2020), it is likely that under high-intensity conditions, both the CeA→NTS and PVN→NTS neurons are recruited. This dual activation may produce not only a sympathetic pressor response but also a marked increase in muscle vascular resistance, thereby limiting the muscle blood flow required for sustaining exercise.

Contrarily, although the lesions of the CeA delayed the onset of the blood pressure surge, they did not abolish it entirely. This indicates that exercise limits are not determined by the CeA alone, but rather by a broader network that integrates multiple autonomic centers, including the PVN and NTS. Thus, the CeA–PVN–NTS circuit represents a central network regulating the sympathetic cardiovascular responses and muscle blood flow during exercise, and may constitute one of the key central mechanisms defining the limits of high-intensity endurance performance.

## 6 CeA–PVN–NTS network during high-intensity endurance exercises

To test the hypothesis that the CeA, PVN, and NTS are interconnected in regulating cardiovascular responses during high-intensity exercises, Ezure et al. (2023) conducted a correlation analysis of c-Fos expression in rats (Ezure et al., 2023). Following the protocol of a previous study (Kim et al., 2020), animals were assigned to sedentary, low-intensity treadmill exercise, and high-intensity treadmill exercise groups after 3 days of habituation training. Brain tissue samples were collected after the exercise, and the number of c-Fos-positive cells in the CeA, PVN, and NTS was quantified by immunostaining. These values were then used for intergroup comparisons and correlation analyses between regions.

The c-Fos expression increased with higher exercise intensity in the CeA, PVN, and NTS. Moreover, no statistical correlations were observed among these regions during resting or low-intensity exercise (Figure 1B). By contrast, in the high-intensity exercise group, a positive correlation was found between the number of c-Fos-positive cells in the CeA–NTS and PVN–NTS pairs (Figure 1C).

These findings indicate that the amygdala–hypothalamus–medulla network is dynamically activated in an exercise intensity-dependent manner and may play an important role in regulating the sympathetic cardiovascular responses. Nonetheless, these results are based on the activity correlations inferred from the c-Fos expression patterns. In the future, studies should more directly manipulate these circuits, using approaches such as optogenetics or chemogenetics, to establish the causal links between the neural activity in this network and exercise performance.

## 7 Discussion

In the present review, we focused on the neural substrates of central fatigue, particularly the subcortical brain regions involved in autonomic regulation, during high-intensity endurance exercise.

Increasing evidence has indicated that the limit of exercise endurance performance is not only determined by the somatic nervous system, but also critically involves the autonomic regulation of circulation and the contribution of brain systems related to emotion and motivation. The medullary regions, such as the NTS, CVLM, and RVLM, play key roles in cardiovascular regulation during exercise. The inputs from the hypothalamic PVN and CeA act on these medullary cardiovascular centers, thereby coordinating both the maintenance of whole-body homeostasis and allostatic regulation required to sustain performance under highly stressful conditions during high-intensity endurance exercises. Indeed, the inhibition (or lesion) of the CeA alters the cardiovascular responses and ultimately enhances exercise performance (Tsukioka et al., 2022). These findings suggest that the emotional centers, including the amygdala and hypothalamus, may act as a “brake” to prevent systemic collapse and protect the organism by restricting muscle blood flow and forcing exercise cessation through excessive sympathetic activation.

However, it is essential to note that this network does not function solely as a “brake.” Robust sympathetic activation is indispensable for sustaining high-intensity performance, and the amygdala–hypothalamus–brainstem network is thought to maintain cardiac output and muscle blood flow by enhancing sympathetic activity. In other words, the same network appears to operate in two opposing modes, facilitating athletic performance and imposing exercise limitation for physiological protection. The boundary between these modes (i.e., the “threshold” of sympathetic activation) may play a critical role in determining exercise limits.

Whether this sympathetic threshold can be improved by training is a critical issue in sports science. To address this question, it is necessary to determine whether endurance training (i) shifts the timing of the abrupt rise in blood pressure to a later point during exercise, or (ii) prolongs the duration for which exercise can be sustained after this abrupt increase has occurred.

If the former is the case, it suggests that training delays the onset of excessive sympathetic responses to the same stressor, thereby indicating an elevated sympathetic threshold. In line with this interpretation, previous studies have shown that lesions of the CeA shift the timing of the abrupt blood pressure increase, reflecting a predominance of sympathetic activation over muscle vasodilation, to a later phase of exercise (Tsukioka et al., 2022). This finding raises the possibility that plastic changes within the amygdala–hypothalamus–brainstem network may occur as a central adaptation to training. In addition, peripheral adaptations may also contribute. Enhancements in cardiac output or metabolic efficiency would reduce the magnitude of physiological stress signals elicited at a given exercise intensity, thereby delaying the point at which the sympathetic threshold is reached.

In contrast, if the latter scenario applies, endurance training may enhance tolerance to the exercise-limiting effects of increased peripheral vascular resistance induced by sympathetic overactivation, which restricts muscle blood flow. One possible mechanism underlying this increased tolerance is systemic vasoconstriction, while vasodilation is preserved in the active muscles. This phenomenon, known as functional sympatholysis, is well established to be augmented by endurance training (Saltin and Mortensen, 2012; Mortensen et al., 2014).

Furthermore, athletes are known to exhibit reduced pain sensitivity compared with non-athletes, and such alterations in pain tolerance may also represent a central factor contributing to sustained performance under high sympathetic drive (Geisler et al., 2021).

When considering high-intensity endurance exercise in a natural context, one might envision an animal pursued by a predator. In such situations, two opposing drives come into conflict, specifically the fear of predation if running ceases versus the warning signal that continued exertion may cause a systemic collapse. Given that the amygdala is crucial for processing emotions such as fear, while the hypothalamus plays a central role in homeostasis, the amygdala–hypothalamus–medulla network can be regarded as a neural substrate mediating this internal struggle. In athletes, the same network may contribute to decision-making under extreme stressful conditions, weighing the fear of “losing to the competition” against the risk of homeostatic breakdown, which influences the timing of exercise termination.

As described in the Introduction, the process leading to exercise limitation cannot be attributed to a single central or peripheral factor. Rather, it emerges from the interaction of multiple influences, including metabolic responses to muscle contraction, body temperature increases, and afferent pain signals arising from muscle damage. In the present review, we propose that, during long-distance endurance exercise such as marathon running, the amygdala CeA–hypothalamus PVN–medullary NTS network may link these central and peripheral factors to modulate the onset and progression of exercise limitation. Whether this framework can be extended to shorter-duration, higher-intensity exercise performed over a brief time frame (e.g., a 400-m sprint) remains to be determined and requires further investigation. Future studies in humans and animals that elucidate how emotional and autonomic interventions affect endurance capacity are warranted to advance our understanding of the mechanisms underlying exercise limitation and contribute to the development of novel strategies to enhance performance.
